# Clinical Management of Major Postoperative Bleeding After Bariatric Surgery

**DOI:** 10.1007/s11695-023-07040-0

**Published:** 2024-01-20

**Authors:** Lars Kollmann, Maximilian Gruber, Johan F. Lock, Christoph-Thomas Germer, Florian Seyfried

**Affiliations:** Department of General, Visceral, Transplantation, Vascular, and Pediatric Surgery, Center of Operative Medicine (ZOM), University Hospital Wuerzburg, Würzburg, Germany

**Keywords:** Bariatric surgery, Postoperative bleeding, Sleeve gastrectomy, Gastric bypass

## Abstract

**Introduction:**

Major postoperative bleeding (mPOB) is the most common complication after bariatric surgery. Its intesity varies from self-limiting to life-threatening situations. Comprehensive decision-making and treatment strategies are mandatory but not established yet.

**Methods:**

We retrospectively analyzied our prospectively collected database of our bariatric patients during 2012–2022. The primary study endpoint was major postoperative bleeding (mPOB) defined as hemoglobin drop > 2 g/dl or clinically relevant bleeding requiring intervention (transfusion, endoscopy or surgery). Secondary endpoints were overall complications according to Clavien-Dindo-Classification and comprehensive-complication-index (CCI).

**Results:**

We identified 1017 patients, of whom 667 underwent gastric bypass (GB) and 350 sleeve gastrectomy (SG). Major postoperative bleeding occured in 39 patients (total 3.8%; 5.1% after GB and 2.3% after SG). Patients with mPOB were more often diagnosed with type 2 diabetes (*p* = 0.039), chronic kidney failure (*p* = 0.013) or received antiplatelet drug treatment (*p* = 0.003). The interval from detection to intervention within 24 h was 92.1% (35/39). Blood transfusions were necessary in 20/39 cases (total 51.3%; 45.2% after GB and 75% after SG; *p* = 0.046). Luminal bleeding only occured after GB (19/31; 61.3%), while all mPOB after SG were intraabdominal (*p* = 0.002). Reoperations were performed in 21/39 (total 53.8%; 48.4% after GB and 75% after SG; *p* = 0.067). CCI in patients with mPOB was 34.7 overall, with 31.2 after GB and 47.9 after SG (*p* = 0.005).

**Conclusion:**

The clinical appearance of mPOB depends on the type of surgery with severe bleedings after SG. We suggest a surgery first approach for mPOB after SG and an endoscopy first approach after GB.

**Graphical Abstract:**

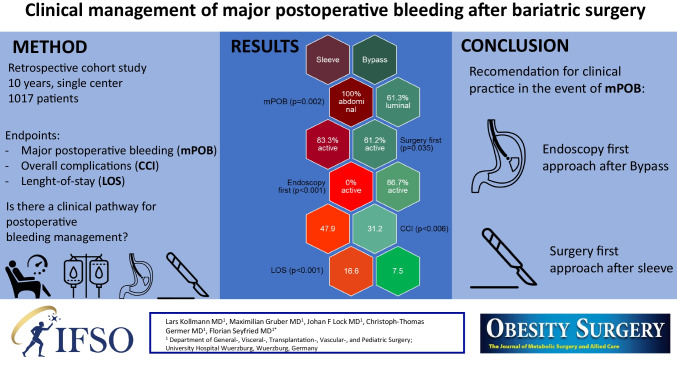

**Supplementary Information:**

The online version contains supplementary material available at 10.1007/s11695-023-07040-0.

## Introduction

Bariatric surgery is the most effective treatment for morbid obesity and its associated comorbidites in the long term, improving patients’ quality of life, socioeconomic status and overal survival [1–4]. Nowadays, bariatric surgery is safely performed at certified centers but still contains the risk for serious complications [5]. The most common reported complication is postoperative bleeding (POB), which occurs in 0.4–4.4% after gastric bypass and in 0.4–3.4% after sleeve gastrectomy (SG), with its intesity varying from self-limiting to severe and life-threatening situations [6–8]. Major POB (mPOB) is the main cause for increased postoperative morbidity and prolonged lengh-of-stay (LOS) in most studies [9]. It is defined by the International Society on Thrombosis and Hemostasis as an event leading to a drop of hemoglobin > 2 g/dl and (1) operative revision or radiological intervention for bleeding control or requiring (2) the postoperative transfusion of ≥ 2 red blood cell packs [10].

Early detection of mPOB can be challenging as the bleeding intensity can vary but can also occur at multiple anatomical sites [9]. In this regard bleeding complications may also vary considerably among the different procedures[6]. Comprehensive decicion making for its early detection and treatment is mandatory but not well established.

The aim of this study was to analyze the clinical presentation and outcomes of mPOB after bariatric surgery and to identify risk factors for mPOB. Secondly, we intented to propose a pathway for comprehensive decision-making and clinical management of mPOB depending on its clinical presentation and the type of surgery.

## Material and Methods

### Patient Collective

We retrospectively analyzed the outcome of all consecutive patients who underwent bariatric surgery at our center from 2012 to 2022. Patients underwent postoperative follow-up in the outpatient clinic 6 weeks after discharge with a rate > 98%. The data was obtained from a prospectively collected database of the national registry (StuDoQ).

All patients with major postoperative bleeding after primary and revisional bypass procedures and sleeve gastrectomies were identified within the prospective database. Major postoperative bleeding was defined as an event leading (1) to a drop of hemoglobin > 2 g/dl AND operative revision or radiological intervention for bleeding control, OR requiring (2) the postoperative transfusion of ≥ 2 red blood cell packs [10].

Patients with mPOB were dichotomized according to the type of surgery being performed (GB vs. SG). Patients receiving conversional GB after prior SG were analyzed within the GB group.

The primary endpoint was major postoperative bleeding. Secondary endpoints were overall complications (Clavien-Dindo Classification and comprehensive complication index, CCI) [11] and length-of-stay (LOS).

### Statistical Analysis

All statistical analyses were performed using IBM SPSS Statistics 29 (International Business Machines Corporation, Armonk, NY). Descriptive data are reported as means with standard deviations, unless otherwise stated. Comparisons between the analyzed cohorts were performed using chi-square test, Fisher’s exact test, Mann–Whitney U-test or a one-way analysis of variance in accordance with data scale and distribution. Linear regressional multivariate analysis were performed. The level of statistical significance was 0.05 (two-sided).

### Surgical Technique

All procedures were performed minimally-invasively in advanced beach chair position by certified bariatric surgeons or residents in training under supervision. All sleeve gastrectomies were stapled using EndoGIA® ultra 60-mm purple linear magazines (Covidien, Dublin, Ireland). In all Roux-en-y gastric bypass procedures the jejuno-jejunostomy was performed using Echelon Flex® linear staplers 60-mm white magazines (Ethicon, Hamburg, Germany). The pouch-gastro-jejunostomy was performed using a 25-mm circular stapler (Ethicon, Hamburg, Germany) with insertion of the anvil via the remnant stomach. The gastric transsection was performed using Echelon Flex® linear staplers 60-mm blue magazines (Ethicon, Hamburg, Germany). The mesenteric gaps were closed by 5-mm stapler devices until 2020, thereafter a running non-resorbable polyfile 2–0 suture was used.

### Postoperative Thrombembolic Prophylaxis

The routine postoperative thrombembolic prophylaxis was obtained with low-molecular-weight heparine (LWMH) in standard dosage for all patients with a body-mass-index (BMI) < 40 kg/m^2^ with 40 mg enoxaparin once daily. All patients with BMI between 40 and 50 kg/m^2^ received 40 mg enoxaparin twice daily and all patients with BMI > 50 kg/m^2^ received 60 mg enoxaparin twice daily. All therapeutic anticoagulated patients received body weight adjusted full dose LMWH under Anti-factor-Xa-level controlling, or in case of chronic renal failure (GFR < 30 ml/min) with unfractioned heparine, according to our local bridging strategy [12]. (Supplement Fig. 1).

#### Bleeding Monitoring and Management

All patients had an overnight stay on a telemetry ward. Thereafter, patient’s vital signs and blood counts were daily monitored for at least 3 days postoperatively. All patients with suspected POB received additional clinical examination, continuous hemodynamic monitoring, repetitive blood count and depending on its clinical presentation an upper gastrointestinal endoscopy (if endoluminal bleeding was suspected) or laparoscopy (if abdominal bleeding was suspected). In unclear situations a contrast-enhanced CT scan or a combination of the above were performed.

## Results

A total of 1017 patients underwent bariatric surgery during 2012 and 2022 and were analyzed. Overall, 350 sleeve gastrectomies (SG), 586 gastric bypass procedures including Roux-en-y and one-anastomosis-gastric bypasses (RYGB, OAGB) and 81 revisional/conversional surgeries were performed. Detailed patient characteristics are provided in Table 1.

**Table 1:** Patients’ and operative characteristics

Patients’ characteristics	Total (*n* = 1017)	Sleeve (*n* = 350)	GB (*n* = 586)	Conversional surgery (*n* = 81)
Sex ratio, No. (M:F)	287:730 (28.2%:71.8%)	127:223 (36.3:63.7)	142:444 (24.2:75.8)	18:63 (22.2:77.8)
Age, mean (SD), y	45 (15–70)	45 (20–70)	44 (17–69)	47 (15–68)
BMI, mean (SD), kg/m^2^	48.4 (21.1–83.1)	54.1 (35.2–83.1)	46.9 (34.1–81)	37.6 (21.1–60.2)
Charlson comorbidity score, mean (SD)	2 (0–10)	2 (0–9)	1 (0–10)	1 (0–6)
ASA classification ≥ III	599 (59.9)	238 (68)	326 (55.6)	45 (55.6)
Edmonton obesity scoring system (EOSS) ≥ 3	292 (28.7)	126 (36)	147 (25.1)	19 (23.5)
Type 2 Diabetes mellitus	462 (45.4)	181 (51.7)	264 (45.1)	17 (21.0)
Insulin-dependent (IDDM)	112 (11.0)	50 (14.3)	59 (10.1)	3 (3.7)
Obstructive sleep apnea (OSAS)	372 (36.6)	158 (45.1)	182 (31.1)	32 (39.5)
Liver steatosis	177 (17.4)	82 (23.4)	86 (14.7)	9 (11.1)
Chronic kidney failure	47 (4.6)	20 (5.7)	26 (4.4)	1 (1.2)
Antiplatelet drug	71 (7.0)	30 (8.6)	36 (6.1)	5 (6.2)
Therapeutic Anticoagulation	52 (5.1)	29 (8.3)	19 (3.2)	4 (4.9)
Perioperative outcome				
Duration of surgery (mean, 95% CI)	89 (42–146)	66 (35–118)	99 (63–155)	113 (53–218)
Postoperative complications (CDC > grade II)	84 (8.6)	21 (6.0)	54 (9.3)	9 (11.1)
Major postoperative bleeding*	39 (3.8)	8 (2.3)	30 (5.1)	1 (1.2)
Intervention: endoscopy	17 (1.7)	1 (0.3)	16 (2.7)	0 (0)
Reoperation for bleeding	21 (2.1)	6 (2)	14 (2.4)	1 (1.2)
Transfusion	21 (2.1)	7 (2)	14 (2.4)	0 (0)
Comprehensive complication index (CCI), (mean 95%CI)	3.9 (0–33.7)	3.7 (0–31)	3.9 (0–33.7)	5.1 (0–36.7)
Length of stay (mean 95% CI)	5 (4–9)	5 (4–9)	5 (4–8)	5 (3–18)

Abbreviations: *GB* gastric bypass, *No.* number, *M* male, *F* female, *SD* standard deviation, *y* years, *BMI* body-mass-index, *CDC* Clavien-Dindo-Classification, *CI* confidence interval, *CCI* comprehensive-complication-index, *IDDM* insuline dependent diabetes mellitus

^*^Defined as reintervention/operation for bleeding or transfusion > 2 EC, or drop of haemoglobin > 2 g/dl

**Fig. 1:**
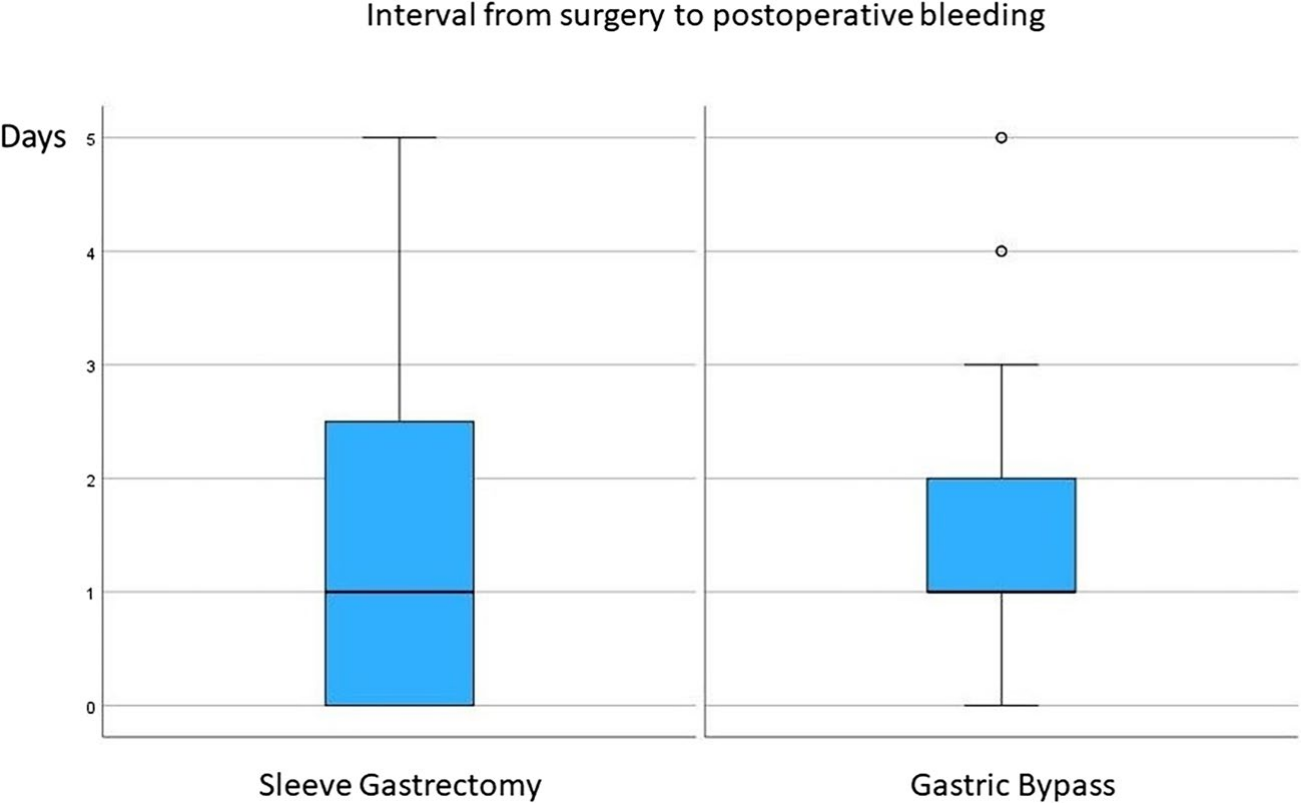
Postoperative interval from surgery to bleeding (in days)

The overall rate for complications Clavien-Dindo grade > II was 84/1017 (8.6%) and 39/1017 (3.8%) for major postoperative bleeding. Thereby, the incidence of mPOB was 8/350 (2.3%) after SG, 38/586 (5.1%) after GB and 1/81 (1.2%) after conversional bariatric surgery (Fig. 1).

Patients with mPOB were more often diagnosed with Type II diabetes mellitus (T2D) (*p* = 0.039), chronic kidney failure (*p* = 0.013) and antiplatelet drug usage (*p* = 0.003) compared to patients without mPOB. However, none of these potential risk factors reached statistical significance in multivariate analysis. (Of note, a trend for chronic kidney failure was found (0.083)).

Patients with mPOB had a higher overall comprehensive complication index (CCI, after Clavien-Dindo-Classification) with 34.7 vs. 2.7 (*p* < 0.001) and longer LOS with 9.4 days vs. 5 days (*p* = 0.045) compared to patients without mPOB. All detailed data are provided in Table 2.

**Table 2:** Patient characteristics overall and major bleeding

	Patients, No. (%)			*p* value
Characteristic	Total (*n* = 1,017)	No bleeding (*n* = 978)	Bleeding (*n* = 39)	
Sex ratio, No. (M:F)	287:730 (28.2:71.8)	273:705 (27.9:71.1)	14:25 (35.9: 64.1)	.277
Age, mean (SD), y	45 (15–70)	45 (15–70)	46 (24–67)	.614
BMI, mean (SD), kg/m^2^	48.4 (21.1–83.1)	49.5 (21.1–83.1)	50.2 (39–72)	.646
Charlson comorbidity score, mean (SD)	2 (0–10)	1.95 (0–10)	2.13 (0–8)	.531
ASA classification ≥ III	599 (59.9)	582 (59.5)	27 (69.2)	.099
Edmonton obesity scoring system (EOSS) ≥ III	292 (28.7)	278 (28.4)	14 (35.9)	.169
Type 2 diabetes mellitus*	462 (45.4)	439 (44.9)	23 (58.9)	.039*
Insulin-dependent (IDDM)	112 (11.0)	105 (10.7)	7 (17.9)	.123
Obstructive sleep apnea (OSAS)	372 (36.6)	362 (37.0)	10 (25.6)	.099
Liver steatosis	177 (17.4)	172 (17.6)	5 (12.8)	.301
Chronic kidney failure*	47 (4.6)	42 (4.3)	5 (12.8)	.013
Antiplatelet drug*	71 (7.0)	67 (6.9)	4 (10.2)	.003**
Oral Anticoagulation	52 (5.1)	51 (5.2)	1 (2.6)	.629
Perioperative outcome				
Duration of surgery (mean, 95% CI)	89 (42–146)	89 (86–91)	91 (81–101)	.689
Postoperative complications (CDC > grade II)	84 (8.6)	52 (5.3)	32 (82.1)	** < .001**
Intervention: endoscopy	27 (2.6)	10 (1.0)	17 (43.6)	** < .001**
Reoperation for bleeding	21 (2.1)	0 (0)	21 (53.8)	** < .001**
Transfusion	20 (2)	0 (0)	21 (53.8)	** < .001**
Comprehensive complication index (CCI), (mean 95%CI)	3.9 (0–33.7)	2.7 (2.1–3.3)	34.7 (29.5–39.7)	** < .001**
Length of stay (mean 95% CI)	5 (4–9)	5 (4–9)	9.4 (7.2–11.6)	**.045**

Abbreviations: *No.* number, *M* male, *F* female, *SD* standard deviation, *y* years, *BMI* body-mass-index, *CDC* Clavien-Dindo-Classification, *CI* confidence interval, *CCI* comprehensive-complication-index, *IDDM* insuline dependent diabetes mellitus

^*^Statistical significance only in univariate, but not in multivariate analysis

* *p*<0.05, ** *p*<0.01

Among patients with mPOB after either gastric bypass procedures or SG the hemoglobin drop from the day of surgery to the first postoperative day was similar (3.9 g/dl). However, the clinical presentation of mPOB was different among the groups. Hematemesis or hematochezia at diagnosis only occurred after GB in (9/31, 29%), but not after SG (*p* = 0.002). Major postoperative bleeding was diagnosed within 48 h after surgery in 29/39 patients (74.4%) overall with 25/31 (80.7%) after GB and 4/8 (50%) after SG (*p* = 0.054). Tachycardia was measured in 16/39 (41.1%) overall with 6/8 (75%) in the SG group and 10/31 (32.3%) in the GB group (*p* = 0.007). The interval from diagnosis to intervention within 24-h rate of the cases was 35/39 (92.1%) overall with 5/8 (62.5%) after SG and 30/31 (96.8%) after GB (*p* = 0.025).

Blood transfusions were overall necessary in 20/39 (51.3%) patients with 6/8 (75%) after SG and 14/31 (45.2%) after GB (*p* = 0.046). Endoscopic interventions were performed in 16/39 (41%) of the cases with 1/8 (12.5%) in the SG group and 15/31 (48.4%) in the GB group (*p* = 0.008). Endoscopy was negative for active or any signs of bleeding in all patients after SG and in 4/15 after GB (26.6%). Reoperations for bleeding were overall performed in 21/39 patients (53.8%) with 6/8 (75%) in the SG group and 15/31 (48.4%) in the GB group (*p* = 0.067). Active bleeding or haemoperitoneum during revisional surgery for mPOB was detected in 5/6 83.3%) patients after SG and in 12/15 patients (80%) after GB.

During revisional surgery for mPOB after SG the staplerline (3/6, 50%) and the short gastric vessles (2/6 (33%) were identified as the bleeding site, while no active bleeding was detected in one patient. During revisional surgery for mPOB after GB the stapler lines of the pouch or small bowel (5/12, 41.7%), the omentum (3/12, 25.0%), and the gastrojejunostomy (2/12, 16.7%) were detected with active bleeding, while hemopertoneum without active bleeding was found in 2/12 (16.7%). Negative laparoscopy occured in 4/16 (25%) patients. Of those, all developed hematochezia during the latter course.

Overall, luminal bleeding (LB) did not occur in the SG group and in 19/31 (61.3%) in the GB group (*p* = 0.002). Extraluminal bleeding (ELB) occured in 20/39 (51.3%) overall with 8/8 (100%) in the SG group and 12/31 (38.7%) in the GB group (*p* < 0.001).

CCI of mPOB patients was 47.9 in the SG and 31.2 in the GB group (*p* = 0.005), the LOS for patients diagnosed with mPOB was 9.4 days overall, with 16.6 days after SG and 7.5 days after GB (*p* < 0.001). One mPOB patient after sleeve gastrectomy developed intermittent multiorgan failure due to hemorhagic shock followed by full recovery (Clavien Dindo IVb). There was no mortality.

## Discussion

The aims of this study were to analyze the clinical course and outcomes of severe postoperative bleeding after bariatric surgery to identify risk factors and to propose a pathway for comprehensive decicion making and clinical management.

Our mPOB with 3.8% was comparable to previous reports from similar institutions [13].

Of note, our patient collective is considerably older, more obese and more often diagnosed with severe obesity-associated comorbidities compared to the international benchmark [14]. Further comparison of the baseline charateristics of our bariatric patients revealed that patients who underwent sleeve gastrectomy had a higher BMI and more severe comorbidities compared to patients who underwent gastric bypass procedures. We need to admit that our sleeve gastrectomy patient collective is not matched neither for patient’s body mass index nor for obesity related comorbidities to our Roux-en-Y gastric bypass group. Despite our sleeve gastrectomy patients had higher BMI and more comorbidities, which has been shown to be an independent risk factor for mPOB [6–8], we found a considerable lower overall mPOB rate. Of note, mPOB after SG was more severe and extraluminal in all cases.

The reason for the overall higher mPOB rate may be attributed to procedure specific details which itself could explain the higher mPOB rate after RYGB. Firstly, the Roux-en-Y gastric bypass was created in an antecolic and antegastric fashion, with the greater omentum and gastro-colic ligament completely divided caudo-cranially using ultrasound scissors. Thus, the divided omentum could become one additional bleeding sites after RYGB.

Secondly, we used a circular stapler to create the gastrojejunostomy, which has been shown to contain a higher risk for endoluminal bleeding compared to a linear anastomosis [9]. The latter could also explain why the mPOB bleeding incidence was surprisingly lower in our revisional bariatric surgery group as in these cases a linear stapler technique for the creation of the gastrojejunostomy was mainly used. Additionally, the creation of the pouch was mainly achieved by transecting the sleeve with only one horizontal linear stapler magazine as the former sleeve gastrectomy made further vertical transection of the stomach unnecessary. This may have reduced the likelihood of stapler line bleedings in our revisional bariatric surgery group.

However, our patients’ characteristics and outcomes are comparable to other tertiary centers [13] (Table 3).

**Table 3:** Patients’ characteristics and outcome major bleeding by procedure

Patients’ characteristics	Total (*n* = 39)	Sleeve (*n* = 8)	GB/conversion (*n* = 31)	*p*-value
Clinical presentation				
Tachycardia	16 (41.1)	6 (75)	10 (32.3)	**.007**
Hypotension	12 (30.8)	8 (100)	4 (12.9)	** < .001**
Hematemesis	9 (23.1)	0 (0)	9 (29)	**.002**
Hematochezia/ Melena	10 (25.6)	0 (0)	10 (32.3)	**.001**
Hemoglobin drop on POD 1 in g/dl (mean 95 and CI)	3.9 (3.3–4.5)	3.8 (2.2–5.4)	4.0 (3.4–4.6)	.729
Postoperative interval bleeding < 48 h (in %)	29 (74.4)	4 (50)	25 (80.7)	.054
Interval bleeding to intervention < 24 h (in %)	35 (92.1)	5(62.5)	30 (96.8)	**.025**
Perioperative outcome				
Postoperative complications (CDC > grade II)	31 (79.5)	6 (75)	25 (80.6)	.369
Grade IIIa	11 (28.2)	0 (0)	11 (35.5)	** < .001**
Grade IIIb	20 (51.3)	5 (62.5)	14 (45.2)	.184
Grade IVb	1 (2.6)	1 (12.5)	0 (0)	.142
Major bleeding:				
Luminal	19 (48.7)	0 (0)	19 (61.3)	**.002**
Abdominal	20 (51.3)	8 (100)	12 (38.7)	** < .001**
Intervention: endoscopy first	16 (41)	1 (12.5)	15 (48.4)	**.008**
Active bleeding*	13 (81.3)	0 (0)	13 (86.7)	** < .001**
No active bleeding	2 (12.5)	0 (0)	2 (13.3)	.064
No bleeding detected	1 (6.25)	1 (100)	0 (0)	.500
Intervention: Surgery first	19 (48.7)	6 (75)	13 (41.9)	**.031**
Active bleeding**	11 (57.9)	5 (83.3)	6 (46.2)	**.035**
No active bleeding	3 (15.8)	1 (16.7)	2 (15.4)	.472
No bleeding detected	5 (26.3)	0 (0)	5 (38.5)	**.002**
Location of bleeding: (in % of abdominal bleeding)				
Staplerline	8 (40)	3 (37.5)	5 (41.7)	.574
Omental	6 (30)	3 (37.5)	3 (25)	.278
Anastomosis	2 (10)	0 (0)	2 (16.7)	.061
Other	4 (20)	2 (25)	2 (16.7)	.328
Localisation bleeding endoscopy: (in % of luminal bleeding)				
Staplerline	5 (26.3)	0 (0)	5 (26.3)	** < .001**
Anastomosis	8 (42.1)	0	8 (42.1)	** < .001**
Jejunojejunostomy	5 (26.3)	0 (0)	5 (26.3)	** < .001**
Transfusion	20 (51.3)	6 (75)	14 (45.2)	**.046**
Management on ICU	14 (35.9)	4 (50)	10 (32.3)	.495
Comprehensive complication index (CCI), (mean 95%CI)	34.7 (29.5 – 39.7)	47.9 (20.9–53.1)	31.2. (20.9 – 47.6)	** < .006**
Length of stay (mean 95% CI)	9.4 (7 – 12)	16.6 (6–31)	7.5 (4–12)	** < .001**
In-hospital mortality	0	0	0	1

Abbreviations: *GB* gastric bypass, *No.* number, *M* male, *F* female, *SD* standard deviation, *y* years, *BMI* body-mass-index, *POD* postoperative day, *CDC* Clavien-Dindo-Classification, *CI* confidence interval, *ICU* intensive care unit, *CCI* comprehensive-complication-index

^*^Percentage from all endoscopies performed

^**^Four negative laparoscopies without active intraabdominal bleeding

In univariate analysis patients with major postoperative bleeding were more often diagnosed with type 2 diabetes, chronic kidney failure, or received antiplatelet drug treatment. Multivariate analysis, however, could not confirm either of these as independent risk factors, with chronic kidney failure showing a strong trend (*p* = 0.084). We assume that our study may be underpowered as diabetes, and chronic kidney failure have been identified as independent risk factors before [15]. In the context of chronic kidney failure and a liberal LMH policy without repetitive measurements of aFXa levels it is possible that LMH accumulated leading to therapeutic aFXa levels with a higher risk for POB.

In contrast, the need for therapeutic anticoagulation was not associated with a higher incidence of POB in our cohort. We assume that these patients may have received more attention upfront surgery with meticulous bridging while surgery may have been performed with more concern regarding hemostasis.

Major postoperative bleeding occurred mainly during the first 48 h after surgery while the interval from detection of bleeding to intervention within 24 h was 92.1% (35/39) overall. However, early intervention for mPOB was more frequent achieved after gastric bypass (96.8%) compared to after sleeve (62.5%). This could be explained by the different clinical appearance of mPOB among the procedures as sings of luminal bleeding only occurred after gastric bypass (29%) prompting to early intervention.

Accordingly, patients with major postoperative bleeding after GB presented with hypotonia and positive shock index in only 12.9%. This is in contrast to a study describing tachycardia and shock symptoms in up to 65% after GB procedures [6, 13, 16].

We assume that the luminal mPOB (61.3%) after GB were detected early and mainly on the day of surgery and were therefore treated immediately while bleedings at the abdominal site after SG were later detected but also more severe. This could have led to a longer active bleeding into the abdominal cavity finally exhausting circulatory compensation.

Endoscopic interventions were performed in 15/31 patients after GB. In four of those, no luminal bleeding was found assuming whether bleeding at the jejuno-jejunostomy (JJ) or extraluminal bleeding. Of note, small bowel obstruction due to clotting of luminal blood at the JJ did not occur but have been described elsewhere [13].

Endoscopic management was successful for active luminal bleedings. We prefer the application of endoscopic clips for POB at the anastomosis or stapler lines and avoid injectional or ablative techniques as they may impair the perfusion of the anastomotic area leading to secondary leakages [17].

During laparoscopic revision for mPOB, we found active bleeding in 87.5% patients after SG but only in 75% after GB group. Transfusion of red blood cell concentrates for mPOB were necessary in 75% after SG but less (45.2%) after GB. Nonsurprising cumulative complication index (CCI) was higher after SG compared to GB.

We need to admit that we have a relatively high rate of revisional surgeries for severe postoperative bleeding compared to previous studies [13]. This may in part be explained by our low threshold for reintervention. Our policy is to act rather early than delay necessary intervention in order to prevent destabilization and life-threatening situations. As a result, blood transfusions were only necessary in 53.8% of our patients, on the other hand a negative surgical intervention was performed in 12.8%.

We use staples with 3.6-mm height for forming the gastric pouch which is widely accepted [18]. For the gastrojejunostomy, we mainly used 25-mm circular staplers with adaptive height.

Stapler lines are not routinely oversewn but reinforced staplers at the subcardial/proximal part during sleeve gastrectomy are used [18]. In severely ill patients or with a non-pausable anticoagulation, we use reinforced staplers for the whole stapling line which has been shown to reduce POB [18].

**Fig. 2:**
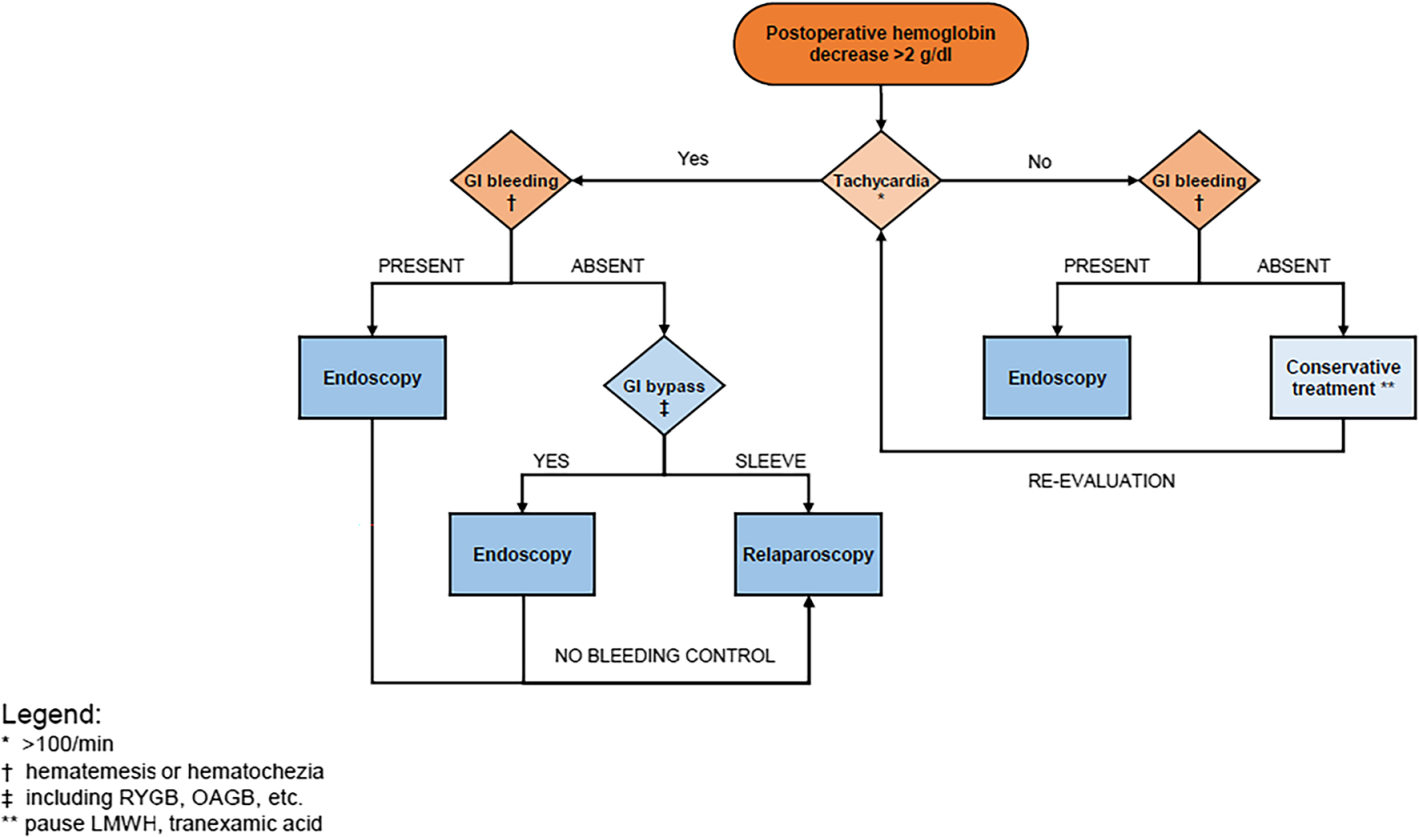
Bleeding management flowchart. Abrreviations: LWMH, low weight molecule heparine; RYGB, roux-en-y-gastric-bypass; OAGB, one-anastomosis-gastric-bypass

We concluded for our clinical management those patients after GB with suspicion of mPOB or with clinical signs of bleeding (oral/rectal) should primarily undergo endoscopic intervention, while patients after SG should undergo laparoscopy (Fig. 2). The goal is, in our opinion, to prevent circulatory decompensation due to bleeding which we mainly achieved. In comparison to other studies, our time to intervention after bleeding is 1 day (or within the next 24 h) compared to up to 6.5 days [13].

There are some limitations to this study due to its single-center retrospective design. Our cohort represents the real-life situation of a German tertiary center with a significant amount of non-benchmark categorized patients.

We also need to admit that mPOB in our cohort is relatively high. This could be explained by our LMWH policy administering relatively high dosages for morbidly obese patients in the perioperative setting with no routine anti-factor Xa monitoring [19]. It has been shown that for bariatric patients a dosage of 60 mg enoxaparine every 12 h may result in therapeutic antiXa-levels [20]. The optimal dosage of LMWH for patients with obesity is still up for discussion^(20)^. On the other hand, we had only one out of 1017 patients with a venous thromboembolism (pulmonary-artery-embolism) within a 6-week-follow-up after bariatric surgery. If a lower level of thrombembolism prophylaxis could have improved our POB remains speculative.

Another point is that only patients with clinically severe POB are reported and, thus, patients with subclinical bleeding or hemoglobin trends are not included.

## Conclusion

Postoperative bleeding is the most frequent severe postoperative complication after bariatric surgery. Major postoperative bleeding occurs more often after GB than after SG. The bleeding site after gastric bypass is endoluminal in more than 60% and therefore clinically more obvious. Major postoperative bleeding after SG is more severe and at the abdominal site. Our data suggest that patients with major POB after SG should be administered to revisional surgery while an endoscopy first approach should be favored for patients after GB. These findings need to be confirmed by multicenter or national registry-based studies.

### Supplementary Information

Below is the link to the electronic supplementary material.

Supplementary file1 (DOCX 167 KB)

## Data Availability

The data is available and can be provided by the corresponding author upon request.

## Abbreviations

(m)POB: (Major) Postoperative bleeding
LOS: Length of stay
GB: Gastric bypass
SG: Sleeve gastrectomy
CCI: Comprehensive complication index
BMI: Body mass index
RYGB: Roux-en-y-gastric bypass
OAGB: One anastomosis gastric bypass
T2D: Diabetes mellitus type II
LB: Luminal bleeding
ELB: Extraluminal bleeding
JJ: Jejuno-jejunostomy
LMWH: Low-molecule-weight-heparine
