# Supplementary material for: Clinical Management of Major Postoperative Bleeding After Bariatric Surgery
Source: Obes Surg. 2024 Jan 20;34(3):751–9. doi: 10.1007/s11695-023-07040-0 (PMC10899369; doi:10.1007/s11695-023-07040-0)
Supplement: Supplementary file 1 — Supplementary file1 (DOCX 167 KB) [file 11695_2023_7040_MOESM1_ESM.docx]

| **Patient** data |
| --- |

**Perioperative anticoagulation in elective surgery**

| **Risk stratification** | | **thromboembolic risk** | | |
| --- | --- | --- | --- | --- |
| **Indication of OAK** |  | **low** | **medium** | **high** |
| 🞎 Atrial fibrillation | \| **CHADS_2_** \| **Score** \| \| --- \| --- \| \| previus stroke \| 2 \| \| age >75 years \| 1 \| \| hypertension \| 1 \| \| diabetes \| 1 \| \| heart insufficieny \| 1 \| \| **sum of points** \| **_____** \| | 🞎 0-2 | 🞎 3-4 | 🞎 5-6  🞎 stroke  <3 months |
| 🞎 Venous thromboembolism (e.g. deep vein thrombosis)  🞎 Pulmonary embolism | |  | 🞎 >3 months | 🞎 ≤3 months |
| 🞎 Heart valve prothesis | 🞎 bileaflet mechanical aortic valve | 🞎 sinus rhythm & CHADS_2_ =0 | 🞎 CHADS_2_ >0 |  |
|  | 🞎 biological aortic valve |  | 🞎 sinus rhythm | 🞎 AF |
|  | 🞎 older aortic mechanical valve |  |  | 🞎 always |
|  | 🞎 tricuspid valve replacement |  |  | 🞎 always |
| 🞎 Thrombophilia | 🞎 antithrombin III deficiency  🞎 antiphospholipid syndrome  🞎 antiphospholipid antibodies  🞎 Factor V Leiden |  | 🞎 always  🞎 always | 🞎 always  🞎 always |
| **Perioperative bridging** | | 🞎 prophylactic LMWH | 🞎 half-dose LMWH | 🞎 full-dose LMWH  🞎 Heparin i.v.* |
| ** in CKD with eGFR <30mL/min with high bleeding and high thromboembolic risk* | | | | |

| **Individual dosing of LMWH (Enoxaparin) s.c.** | | | | | | |
| --- | --- | --- | --- | --- | --- | --- |
| **Age:** ____ years | | | | **Weight:** _____ kg | **eGFR**: _____ mL/min | |
| 🞎 Prophylactic LMWH | | | **40mg g.d** | | | |
| 🞎 half-dose / full-dose LMWH | | **weight-adjusted dose 0.8 – 1.0 mg/kg BW**  🡪 in morbid obesity half-dose split dose e.g. 140kg in 60-70mg b.i.d)  🡪 in elderly (>75 years) better 0.8 mg/kg BW  🡪 if 🞎 CKD with eGFR <50mL/min 50% dose reduction  🡪 check anti–factor Xa level 3-5 days postoperatively | | | | **(max. 100mg single dose)**   |
| **Timing of LMWH bridging**  for warfarin pre- and postoperative briding, for DOAK preoperative interruption and postoperative bridging    ***( )*** *if additional platelet inhibation * if CKD with eGFR <50mL/min* | | | | | | |
| **Preoperative interruption of OAK** | | | | | | |
| 🞎 Warfarin | 🡪 minumum 7 days interruption prior surgery  🡪 INR check every 2 days 🡪 off INR <2 beginn of briding (as stated above)  🡪 check INR the day before surgery  🡪 target INR <1,3 (*no i.v. substitution of vitamin K)* | | | | | |
| 🞎 Apixaban  🞎 Rivaroxaban  🞎 Edoxaban | \|  \| eGFR \| ≥80 \| 50-80 \| 30-50 \| 15-30 \| mL/min \| \| --- \| --- \| --- \| --- \| --- \| --- \| --- \| \| Bleeding risk*: \| low \| 🞎 ≥24h \| 🞎 ≥24h \| 🞎 ≥24h \| 🞎 ≥36h \|  \| \| medium/ high \| 🞎 ≥ 48h \| 🞎 48-72h \| 🞎 72-96h \| 🞎 ≥96h \|  \| | | | | | |
| 🞎 Dabigatran | \|  \| eGFR \| ≥80 \| 50-80 \| 30-50 \| mL/min \| \| --- \| --- \| --- \| --- \| --- \| --- \| \| Bleeding risk*: \| low \| 🞎 ≥24h \| 🞎 ≥36h \| 🞎 ≥48h \|  \| \| medium/ high \| 🞎 ≥48h \| 🞎 ≥60h \| 🞎 ≥96h \|  \| | | | | | |

Abbreviations: OAK: oral anticoagulation; AF: atrial fibrillation; LWMH: low molecule weight heparine; CKD: chronic kidney disease; GFR: glomerulary filtration rate; s.c.: subcutaneous; BW: body weight; DOAK: dual oral anticoagulation; INR: international normalized ratio; i.v. intravenous.
